# Cognitive and Motor Function Effects of Antipsychotics in Traumatic Brain Injury: A Systematic Review of Pre-Clinical Studies

**DOI:** 10.1089/neur.2023.0108

**Published:** 2024-03-05

**Authors:** Gabrielle Cataford, Laurie-Anne Monton, Stephanie Karzon, Camille Livernoche-Leduc, Mar Saavedra-Mitjans, Marie-Julie Potvin, Francis Bernard, Lisa Burry, Caroline Arbour, David R. Williamson

**Affiliations:** ^1^ Faculté de pharmacie, Université de Montréal, Montreal, Quebec, Canada.; ^2^ Faculté de Médecine, Université de Montréal, Montreal, Quebec, Canada.; ^3^ Départment de psychologie, Université du Québec à Montréal, Montreal, Quebec, Canada.; ^4^ Research center, CIUSSS-Nord-de-l'Île-de-Montréal, Hôpital du Sacré-Cœur de Montréal. Montreal, Quebec, Canada.; ^5^ Pharmacy Department, Mount Sinai Hospital. Toronto, Ontario, Canada.; ^6^ Leslie Dan Faculty of Pharmacy, University of Toronto, Toronto, Ontario, Canada.; ^7^ Faculté de sciences infirmières, Université de Montréal, Montreal, Quebec, Canada.

**Keywords:** antipsychotics, behavior, cognition, functional recovery, pre-clinical model, traumatic brain injury

## Abstract

Traumatic brain injury (TBI) survivors often suffer from agitated behaviors and will most likely receive pharmacological treatments. Choosing an optimal and safe treatment that will not interfere with neurological recovery remains controversial. By interfering with dopaminergic circuits, antipsychotics may impede processes important to cognitive recovery. Despite their frequent use, there have been no large randomized controlled studies of antipsychotics for the management of agitated behaviors during the acute TBI recovery period. We conducted a systematic review and meta-analysis of pre-clinical studies evaluating the effects of antipsychotics post-TBI on both cognitive and motor recovery. MEDLINE and Embase databases were searched up to August 2, 2023. Pre-clinical studies evaluating the effects of antipsychotics on cognitive and motor functions post-TBI were considered. Risk of bias was evaluated with the Systematic Review Centre for Laboratory Animal Experimentation (SYRCLE) tool. We identified 15 studies including a total of 1188 rodents, mostly conducted in male Sprague-Dawley rats using cortical impact injury. The analysis revealed no consistent effect of haloperidol on motor functions, but risperidone was associated with a significant impairment in motor function on day 5 post-injury (7.05 sec; 95% confidence interval [CI]: 1.47, 12.62; I^2^ = 92%). Other atypical antipsychotics did not result in impaired motor function. When evaluating cognitive function, haloperidol- (23.00 sec; 95% CI: 17.42–28.59; I^2^ = 7%) and risperidone-treated rats (24.27 sec; 95% CI: 16.18–32.36; I^2^ = 0%) were consistently impaired when compared to controls. In studies evaluating atypical antipsychotics, no impairments were observed. Clinicians should avoid the regular use of haloperidol and risperidone, and future human studies should be conducted with atypical antipsychotics.

## Introduction

Traumatic brain injury (TBI) is a leading cause of mortality and disability worldwide. In the United States alone, ∼50,000 persons die each year from TBI and >5 million live with TBI-related disabilities.^1,2^ Depending on injury severity, TBI survivors will often suffer from agitated behaviors during their hospital recovery and receive pharmacological treatment to prevent self-harm or harm to others.^3–6^ However, choosing an optimal and safe pharmacological treatment that will not interfere with neurological recovery remains controversial.^7,8^

Agitation can be defined as a state of confusion during the period of impaired consciousness that follows the initial brain injury and is characterized by excessive behaviors such as emotional unrest, akathisia, impulsivity, disorganized thinking or disinhibition, and aggression.^9^ In some cases, agitation can develop or persist after emergence from the state of impaired consciousness.^10^ Agitation occurs in 52–57% and 20–41% of brain-injured patients in the intensive care unit (ICU) and acute care unit, respectively.^11–15^ The pooled prevalence of agitation is of 31.7% during inpatient care and 32.2% during rehabilitation.^10^ Neurotransmitter and autonomic nervous system imbalances are among the potential causes and are the targets for pharmacological interventions.^16,17^ During a hospital stay, patients with a TBI are often treated with antipsychotics to manage agitation, aggression, or other challenging behaviors.^3–6,18,19^ Despite their frequent use, antipsychotics for the management of agitation in TBI patients have not been adequately studied in a controlled setting.^7^ To date, only two small pilot randomized controlled studies have evaluated olanzapine and risperidone.^20,21^ Moreover, by interfering with dopaminergic circuits, antipsychotics may impede neuronal plasticity processes important to cognitive recovery.^22,23^

Observational studies in TBI patients have also suggested longer post-traumatic amnesia recovery with first-generation antipsychotics such as haloperidol.^24,25^ Studies have shown the potential of amantadine, a dopamine agonist, to improve functional outcomes in TBI.^26,27^ This further highlights the controversy of dopamine receptor blockade as a treatment for agitation in that context. Based on these data, TBI rehabilitation guidelines have suggested short-term use only.^28,29^

To date, there has been no systematic evaluation of the pre-clinical literature summarizing the effects of antipsychotics on cognitive and motor function after experimental TBI. Therefore, we conducted a systematic review of pre-clinical studies evaluating the effects of antipsychotics post-TBI on both cognitive and motor recovery. The objective was to systematically review, summarize, and appraise the literature addressing the effect of antipsychotics after a TBI on cognitive and motor function in animal models.

## Methods

The systematic review was carried out in accordance with the Preferred Reporting Items for Systematic Reviews and Meta-Analyses (PRISMA) guidelines and the Systematic Review Centre for Laboratory Animal Experimentation (SYRCLE) guidelines for animal intervention studies. The protocol was registered on the International Platform of Registered Systematic Review and Meta-analysis Protocols (INPLASY202310034).(30)

### Search methods and data sources

The search strategy aimed at identifying all eligible studies regardless of publication status. Two investigators (D.W., S.K.), with the help of a health sciences librarian with expertise in systematic reviews, designed the search strategy and conducted the search (Supplement S1).^31^ The MEDLINE and Embase databases were searched from inception to August 2, 2023 with the following search terms: craniocerebral trauma, cognition and consciousness, translational medical research, and antipsychotics agents. In addition, references of identified studies were screened for additional studies. Studies of all languages were considered for inclusion.

### Data collection and analysis

After the literature search, an EndNote database (EndNote version X9 Thomson Reuters, New York) was created to manage citations. Two independent authors (D.W., S.K.) screened the titles and/or abstracts of each identified publication for eligibility. Eligible citations were read in the full-text version by two independent authors (D.W., S.K.) and evaluated for inclusion using the eligibility criteria, which were based on type of study, participant characteristics, interventions, and outcome measures. In the case of disagreements, they were resolved by consensus and discussion with a third reviewer (G.C.). The selection process was documented using a PRISMA flow diagram.

### Data extraction and management

Data from all included studies were independently extracted in duplicate using a pre-tested data extraction form by the authors (G.C., S.K.). The following variables were recorded for each study: study title, first author, publication year, country of origin, language of publication, source of funding, pre-clinical animal model, experimental TBI method, outcome measures for cognitive, functional, behavioral, and motor evaluation, and pharmacological intervention, including dose and timing of medication administration, type of comparator, and timing of evaluations.

### Types of studies

All pre-clinical animal studies evaluating the effects of antipsychotics on cognitive and motor functions were considered for inclusion. We only included studies administrating antipsychotic agents after inducing a TBI. Studies, including randomized and non-randomized experiments, comparing pharmacological agents to a placebo, an active treatment, or a non-pharmacological intervention were included.

### Types of participants

We considered studies with all types of mammalians reproducing any form of TBI, including focal, diffuse, and complex TBIs induced by blast injury, penetrating injury, repetitive injury, or other types of models. Common models include weight-drop, fluid percussion injury, blast injury, and controlled cortical impact injury models.^32^ There were no restrictions with respect to sex, age, or mammalian species.

### Interventions and comparators

This systematic review evaluated any antipsychotic, either first or second generation, administered *in vivo* after a TBI. Studies comparing these pharmacological agents to a placebo, an active treatment, or a non-pharmacological intervention were considered for inclusion.

### Outcome measures

We included animal models measuring motor functions (such as beam-balance task and beam-walk time), cognitive functions (such as the Morris water maze [MWM]), and histological outcomes. No restrictions with respect to timeline of outcome measures were imposed. The beam-balance task consists of placing the rat on an elevated narrow wooden beam and recording the duration it remains on it for a maximum of 60 sec. This tool evaluates vestibulomotor function and balance. The amount of time the animal balances on the beam is recorded. The beam-walk task assesses refined locomotor activity and consists of training/assessing animals using a negative-reinforcement paradigm to escape ambient light and high decibel white noise by traversing an elevated narrow wooden beam and entering a darkened goal box at the opposite end.^33^

Performance is assessed by the latency to traverse the beam. The beam-walk score uses criteria for distance traveled based on a rating scale of 0–5, where 0 indicates an inability to ambulate beyond the start point, 1–4 corresponds to distal segments of 20, 40, 60, or 80 cm from the starting point, respectively, and 5 indicates traversal of the entire length of the beam and entrance into the goal box.^34^ The MWM task assesses spatial learning and consists in rodents identifying a submerged escape platform within a plexiglass swimming pool.^35^ The MWM task measures time to locate the platform when submerged below the water surface. As part of the MWM task, memory retention is assessed by removing the platform from the pool, placing the rodents in the maze, letting them freely explore during 30 sec, and measuring the proportion of time spent exploring the target quadrant.^36^ To evaluate the safety of antipsychotics, we examined the post-mortem histological effects of antipsychotics on cortical lesion size and neuron morphology.

### Quality assessment

Three reviewers (G.C., L.A.M., and D.W.) independently evaluated each included study with the SYRCLE's risk-of-bias tool for animal studies.^30^ The SYRCLE's risk-of-bias tool is an adapted version of the Cochrane risk-of-bias tool. In cases of disagreement regarding the risk of bias, a fourth reviewer was consulted to resolve the issue (C.L.L.). The SYRCLE's risk-of-bias tool assesses the quality of studies according to 10 domains: sequence generation, baseline characteristics, allocation concealment, random housing, blinding, random outcome assessment, incomplete outcome data, selective reporting, and other sources of bias. This tool assigns each item mentioned in the study to one of three categories of bias: low risk, high risk, or unclear risk. As suggested, the results of individual studies were presented in a table.

### Analysis

The results of the systematic review are presented as both a descriptive overview and meta-analysis. To enable meta-analysis, we graphically extrapolated repeated data measures and standard errors of the mean using WebPlotDigitizer 4.3 of the following continuous variables: beam-balance time, beam-walk time, beam distance, MWM, and visual platform performance. The analysis was stratified by type of antipsychotic (haloperidol, risperidone, and other atypical antipsychotics) using the groups with continuous daily drug administration. Risperidone was analyzed separately given its high-affinity D2-blocking properties. When more than one drug dose was studied, we analyzed the group with the lowest dose given the relatively high doses administered to animals. In a study comparing males and females, we included both groups separately. In studies evaluating the effects of environmental enrichment in the presence of antipsychotics, we included animals exposed to vehicle and antipsychotics in both exposed and non-exposed to environmental enrichment. For the daily evaluation of beam-balance task and beam-walk time, given that the maximum is reached at 60 sec, we excluded study results when the maximum was reached in one of the groups. For beam-balance task and beam-walk time, we also analyzed means over days 1–5.

Means and standard deviations were calculated using the method described by Higgins and colleagues.^37^ Because the timing of MWM measurements varied from day 1 to day 26 post-injury, we combined measurements closest to day 18, which was the day with the most measurements. In secondary analyses, we evaluated the effects of different doses of antipsychotics compared to intermittent dosing of antipsychotics.

If the statistical heterogeneity was acceptable (I^2^ < 50%), we proceeded to a meta-analysis of the data using Review Manager (RevMan 5.4) software (Nordic Cochrane Center, Cochrane Collaboration).^38^ A random-effects model was used if at least two studies were available. The continuous results are presented with 95% confidence intervals (CIs). Categorical results are presented as odd ratios with 95% CI.

## Results

**FIG. 1.**
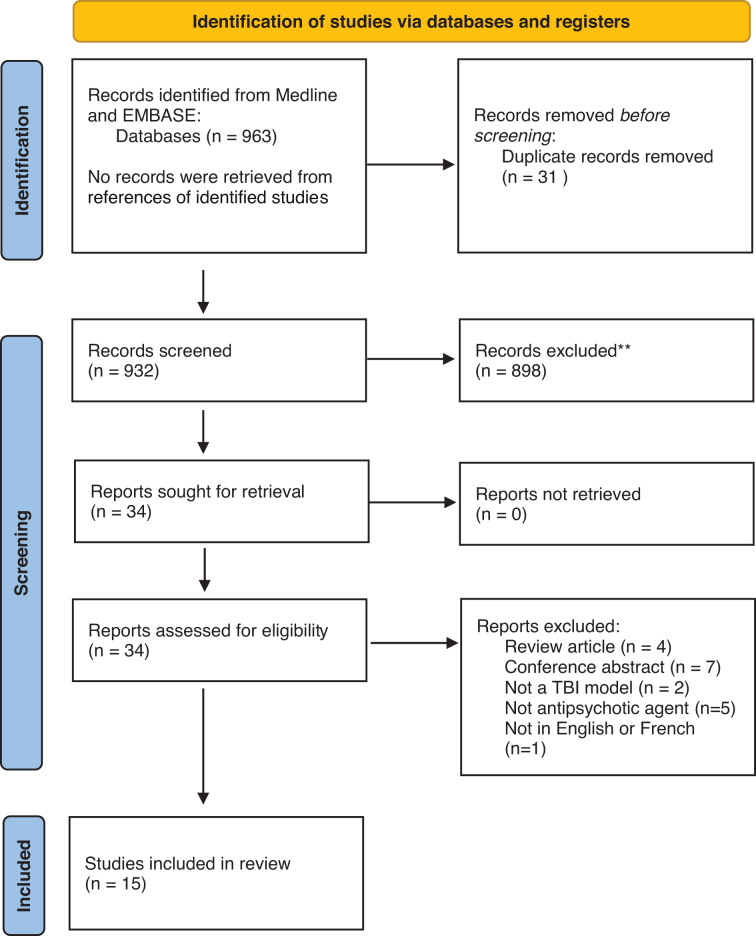
PRISMA flow diagram.

The search strategy identified 963 citations (Fig. 1). After removal of duplicates, 935 citations were screened by examining the title and abstract. Full-text assessment of 34 studies identified a total of 15 studies meeting the inclusion and exclusion criteria. Studies were mainly excluded because they were a conference abstract (*n* = 7) or a review article (*n* = 4) or because they studied other agents than antipsychotics (*n* = 5) or other non-TBI disease models (*n* = 2).

### Study characteristics

In total, 1188 rodents were included in the studies, with sample sizes ranging from 40 to 194 rodents (Table 1). The majority of studies were from the United States and conducted in male Sprague-Dawley rats using cortical impact injury as the TBI method.^33,34,36,39–46^ Only two studies used a drop-weight mild TBI model^47,48^ whereas one used a lateral fluid percussion injury^49^ or craniocervical whiplash injury.^50^ Haloperidol was the most studied antipsychotic (10 studies),^33,39,41–47,49^ followed by risperidone (three studies),^43–45^ sulpiride (a D2-specific antagonist; two studies),^48^ and aripiprazole (two studies).^36,40^ Quetiapine,^46^ olanzapine,^49^ levomepromazine (also known as methotrimeprazine),^50^ and SCH-23390 (an experimental D1-specific antagonist)^48^ were each evaluated in one study. Post-TBI, the agents were typically administered daily for a duration of 19 days starting 24 h after post-injury,^33,34,36,39–43,45,46^ although a few studies evaluated the effects of alternate day dosing or of a single dose.^44,46–50^

**Table 1.** Study Characteristics

Authors (country)	Design (year)	Total sample size	Model	TBI method	Comparator	Antipsychotic, route of administration, dose, and duration	Conditions
Bao et al. (USA)	RCT (2019)	84	Male Sprague-Dawley rats	Cortical impact injury	Vehicle 1 mL/kg	Haloperidol 0.5 mg/kg i.p. daily for 19 days	Environmental enrichment
Besagar et al. (USA)	RCT (2019)	52	Male Sprague-Dawley rats	Cortical impact injury of moderate severity	Vehicle 1 mL/kg	Aripiprazole 0.1 mg/kg i.p. daily for 19 days	Environmental enrichment
Boismare et al. (France)	RCT (1978)	40	Long Evans female rats	Craniocervical injury whiplash	Diazepam 1 mg/kg i.p.	Levomepromazine 1 mg/kg i.p. for one dose 1 h post-TBI	
Carlson et al. (USA)	RCT (2018)	56	Male Sprague-Dawley rats	Cortical impact injury of moderate severity	Vehicle 1 mL/kg	Risperidone 0.45 mg/kg i.p. 1 × , 3 × , or 7 × per week until day 19	
Folweiler et al. (USA)	RCT (2017)	60	Male Sprague-Dawley rats	Cortical impact injury	Vehicle 1 mL/kg	Haloperidol 0.5 mg/kg i.p. daily for 19 days	Environmental enrichment
Free et al. (USA)	RCT (2017)	72	Male and female Sprague-Dawley rats	Cortical impact injury	Vehicle 1 mL/kg	Haloperidol 0.5 mg/kg i.p. daily for 19 days	
Hoffman et al. (USA)	RCT (2008)	54	Male and female Sprague-Dawley rats	Cortical impact injury	Vehicle 1 mL/kg	Haloperidol 0.5 mg/kg or risperidone 0.45 mg/kg i.p. daily for 19 days	
Kline et al. (USA)	RCT (2007)	60	Male Sprague-Dawley rats	Cortical impact injury	Vehicle 1 mL/kg	Haloperidol 0.5 mg/kg Riperidone 0.045 mg/kg Riperidone 0.45 mg/kg Riperidone 4.5 mg/kg, i.p. in two phases (one dose after 24 h, after 19 days once-daily for 5 days)	
Kline et al. (USA)	RCT (2008)	65	Male Sprague-Dawley rats	Cortical impact injury	Vehicle 1 mL/kg	Haloperidol 0.5 mg/kg Riperidone 0.045 mg/kg Riperidone 0.45 mg/kg Riperidone 4.5 mg/kg, i.p. daily for 19 days	
Phelps et al. (USA)	RCT (2015)	60	Male Sprague-Dawley rats	Cortical impact injury	Vehicle 1 mL/kg	Haloperidol 0.5 mg/kg Risperidone 0.45 mg/kg, i.p. daily for 19 days	
Phelps et al. (USA)	RCT (2017)	40	Male Sprague-Dawley rats	Cortical impact injury	Vehicle 1 mL/kg	Aripiprazole 0.1 mg/kg Aripiprazole 1.0 mg/kg	
Tang et al. (a) (Japan)	Controlled study (1997)	194	Male ddY mice	Drop-weight mild TBI	Vehicle 1 mL/kg	Haloperidol 0.3 mg/kg Haloperidol 1 mg/kg Haloperidol 3 mg/kg, i.p. 15 min post-TBI	
Tang et al. (b) (Japan)	Controlled study (1997)	181	Male ddY mice	Drop-weight mild TBI	Vehicle 1 mL/kg	Sulpiride 3 mg/kg Sulpiride 30 mg/kg SCH-23390 0.03 mg/kg SCH-23390 0.30 mg/kg + combinations	
Weeks et al. (USA)	RCT (2018)	70	Male Sprague-Dawley rats	Cortical impact injury	Vehicle 1 mL/kg	Haloperidol 0.5 mg/kg Quetiapine (10 mg/kg), i.p. daily, once-daily, or every other day	
Wilson et al. (USA)	RCT (2003)	100	Male Sprague-Dawley rats	Lateral fluid percussion injury	Vehicle 1 mL/kg	Haloperidol 0.03 mg/kg Haloperidol 0.10 mg/kg Haloperidol 0.30 mg/kg Olanzapine 0.3 mg/kg Olanzapine 1.0 mg/kg Olanzapine 3.0 mg/kg, i.p. daily from post-TBI days 1–15	

RCT, randomized controlled trial; TBI, traumatic brain injury; i.p., intraperitoneally.

### Quality of evidence

Quality of evidence was evaluated using the SYRCLE risk-of-bias tool for animal studies (Supplementary Table S1). Overall, most studies were at low risk of bias, but certain domains were not reported by some or all of the studies. Although most often described as randomized, the investigators did not describe the sequence generation process and maintenance of allocation concealment was impossible to assess in 13 of 15 studies (86,7%). The selection of animals at random for outcome assessment was never mentioned, and assessment of the blinding of the outcome assessor was also difficult to assess in 3 of 15 studies (20%).

### Motor performance

Motor performance was most often evaluated using the beam-balance and beam-walk tasks.^33,34,36,39–46,49^

#### Beam-balance task

In the eight studies evaluating haloperidol, all but one studied an intraperitoneal dose of 0.5 mg/kg and the beam-balance task was evaluated between days 1 and 5 post-injury. In this test, longer durations indicate better performance.^46^ When comparing the nhaloperidol group to the vehicle group, meta-analysis of the seven studies with available data showed no difference in beam-balance on day 1 (−5.16 sec; 95% CI: −11.45, 1.14) and day 3 (−2.41 sec; 95% CI: −6.83, 2.00), but a statistically significant difference (−8.53 sec; 95% CI: −14.02, −3.04) in favor of the vehicle group on day 2 (Table 2).^33,39,41,42,44–46^ Meta-analysis of days 4 and day 5 combined six and four studies, respectively, and showed no differences (Table 2). The analysis of combined mean beam-balance times from days 1 to 5 from eight studies also showed no difference (−2.81 sec; 95% CI: −7.23, 1.61). In one study evaluating the effects of three different doses of haloperidol (0.03, 0.1, and 0.3 mg/kg) compared to the vehicle over 5 days, the haloperidol groups showed shorter durations of the beam-balance task, but the difference was not statistically different.^49^ In studies evaluating the beam-balance task beyond 5 days, no difference was found after 20, 26, 48, and 108 days.^44,45^

**Table 2.** Meta-Analyses of Motor Performance

Drug	Studies	Participants	Mean difference (sec)	I_2_
Motor performance: beam balance				
Haloperidol day 1	7	214	–5.16 [95% CI −11.45, 1.14]	42%
Haloperidol day 2	7	214	–8.53 [95% CI −14.02, −3.04]	14%
Haloperidol day 3	7	194	–2.41 [95% CI −6.83, 2.00]	0%
Haloperidol day 4	6	148	–1.02 [95% CI −4.99, 2.95]	37%
Haloperidol day 5	4	104	–2.41 [95% CI −6.69, 1.87]	54%
Haloperidol days 1–5	8	234	–2.81 [95% CI −7.23, 1.61]	0%
Risperidone day 1	3	62	–6.97 [95% CI −21.12, 7.19]	54%
Risperidone day 2	3	62	–7.83 [95% CI −31.45, 15.78]	0%
Risperidone days 1–5	3	62	–1.92 [95% CI −11.50, 7.66]	14%
Motor performance: beam walk				
Haloperidol day 3	8	208	5.14 [95% CI −5.85, 16.14]	88%
Haloperidol day 4	8	234	4.69 [95% CI −3.02, 12.39]	61%
Haloperidol day 5	8	234	10.28 [95% CI 3.05, 17.50]	80%
Haloperidol days 1–5	9	254	2.66 [95% CI −1.15, 6.48]	0%
Risperidone day 3	5	102	11.51 [95% CI −5.42, 28.43]	84%
Risperidone day 4	5	102	10.78 [95% CI −2.11, 23.67]	84%
Risperidone day 5	5	102	7.05 [95% CI 1.47, 12.62]	92%
Risperidone days 1–5	5	102	5.79 [95% CI −4.12, 15.71]	28%

CI, confidence interval.

When comparing risperidone 0.45 mg/kg to the vehicle group, the meta-analysis of the three studies showed no difference in beam-balance on day 1 and day 2 (Table 2).^33,44,45^ No meta-analysis was carried out for risperidone on days 3, 4, and 5 because most groups had reached the 60-sec limit. The analysis of combined mean beam-balance times from days 1 to 5 from three studies also showed no difference (−1.92 sec; 95% CI: −11.50, 7.66). In one study, the administration of daily injections of risperidone 4.5 mg/kg on days 19–23 post-TBI was significantly associated with motor impairment compared to the vehicle, risperidone 0.045, risperidone 0.45, and haloperidol 0.5 mg/kg groups (*p* < 0.05).^44^ In another study, a *post hoc* analysis showed that risperidone 0.45 mg/kg–exposed animals performed significantly worse than vehicle groups over the 5 study days (*p* = 0.0002).^33^ When evaluating aripiprazole (0.1 and 1 mg/kg) over days 1–5 post-injury, only the 1-mg/kg group performed significantly better than the vehicle group (*p* < 0.05).^36^ Beam-balance times over days 1–5 post-injury with olanzapine and quetiapine were not different than the vehicle group.^46,49^

#### Beam-walk task

In eight studies evaluating the effect of haloperidol on the beam-walk task, meta-analyses of days 3 and 4 showed no statistically significant differences (Table 2). In this task, shorter durations indicate better performance. A significant impairment in traversal times for animals exposed to haloperidol 0.5 mg/kg was noted on day 5 post-injury compared to the vehicle group (Table 2). The analysis of combined mean beam-walk times from days 1 to 5 from eight studies also showed no difference (−2.81; 95% CI: −7.23, 1.61). When comparing three doses (0.03, 0.1, and 0.3 mg/kg) of haloperidol over days 1–5 post-injury, injured groups showed impaired performance compared to the sham-injured group, but the was no difference between animals treated with haloperidol and the vehicle.^49^ In five studies evaluating the effects of risperidone 0.45 mg/kg, a significant impairment in traversal times for animals exposed to risperidone was also noted on day 5 post-injury (Table 2).^33,34,43–45^ No difference was noted on days 3 and 4 (Table 2). When combining the four studies that administered risperidone daily rather than only once post-TBI, there was also a significant difference on day 4 (14.95 sec; 95% CI: 1.25, 28.65).^33,34,43,45^ However, the analysis of combined mean beam-balance times from days 1 to 5 showed no difference (5.79; 95% CI: −4.12, 15.71). When combining the two aripiprazole studies, no difference was found on days 1–5.^36,40^ Beam-walk times over days 1–5 post-injury with olanzapine and quetiapine were not different than the vehicle.^46,49^ In studies evaluating the beam-walk task beyond 5 days, no difference was found after 20, 26, 48, and 108 days.^44,45^

A total of four studies evaluated beam-walk scores with three different agents (haloperidol, risperidone, and aripiprazole).^34,39,40,42^ In one study, the haloperidol-treated females performed better than the haloperidol-treated males.^42^ Additionally, the haloperidol-treated males were more impaired on the last day of testing.^42^ The daily risperidone-treated animals were significantly impaired in comparison to the vehicle and risperidone three times a week groups (*p* < 0.05).^34^ Interestingly, aripiprazole-treated animals exhibited a better beam-walk score relative to the vehicle group (*p* < 0.05).^40^

### Cognitive function

Cognitive function was mostly assessed using the MWM and probe trials, which assess spatial learning and memory retention and are sensitive to cognitive dysfunction post-TBI.

#### Morris water maze

**FIG. 2.**
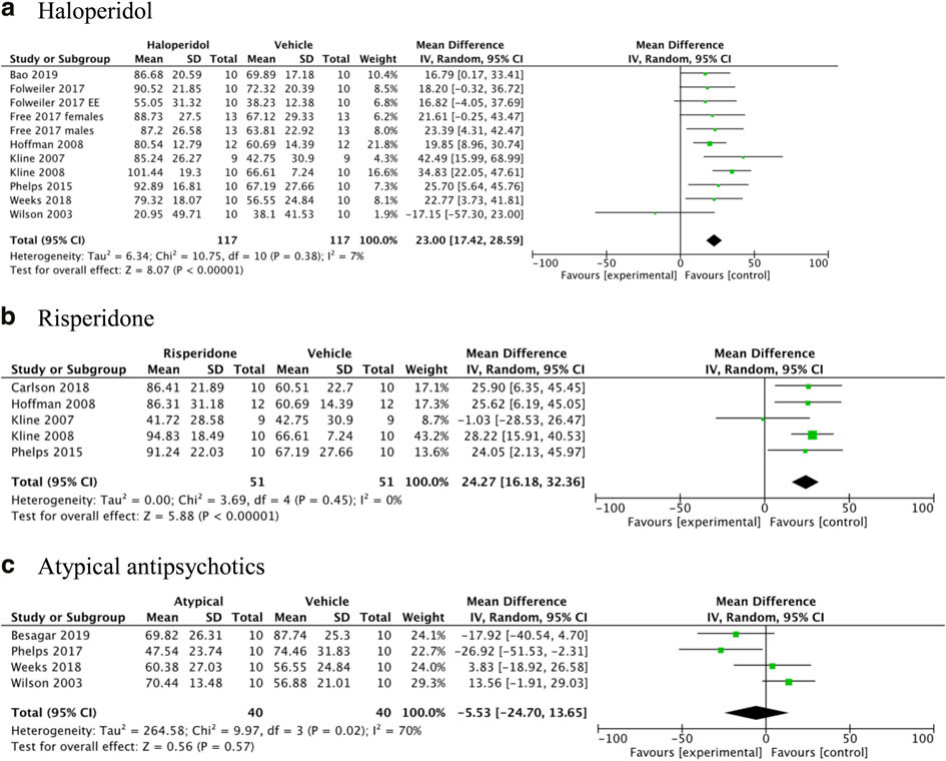
Cognitive function: Morris water maze.

In the nine studies evaluating the MWM with haloperidol, the haloperidol-treated animals were significantly more impaired than the vehicle controls after 15–18 days (Fig. 2A). The five studies of risperidone showed similar results with increased impairment in the risperidone-treated animals after 15–18 days (Fig. 2B). In one study, the effects of haloperidol administered during 19 consecutive days on the MWM task persisted 1 month after drug cessation,^45^ whereas the risperidone-exposed group did show some improvement 1 month after drug cessation and was not statistically different than the vehicle group.^51^ The combination of other atypical antipsychotics (olanzapine, quetiapine, and aripiprazole) showed no increased impairment after 15–18 days (Fig. 2C). When combining the two studies evaluating aripiprazole and including a group with environmental enrichment, there was a trend toward a reduction in MWM impairment (−13.27; 95% CI: −30.31, 3.77; I^2^ = 42%). Visual platform placement at day 19 post-injury was not impeded by haloperidol (four studies) or risperidone (two studies; Table 2).

#### Probe trial

**FIG. 3.**
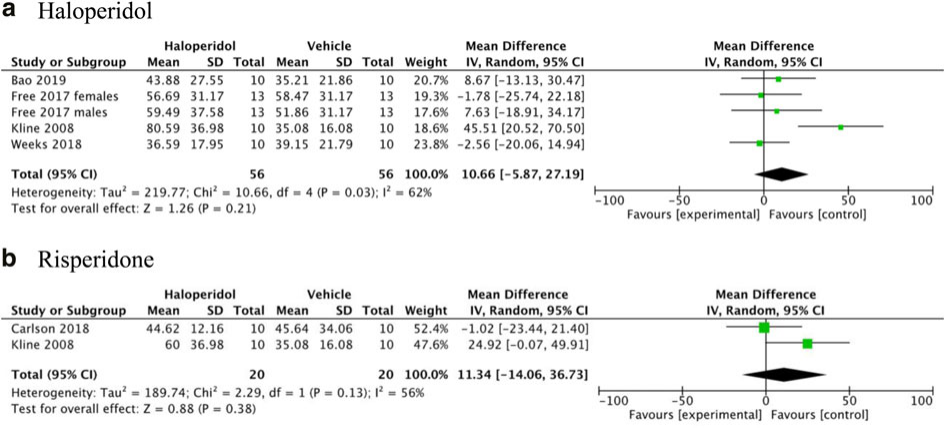
Cognitive function: visual platform performance.

Memory retention was assessed in a total of seven studies evaluating haloperidol, aripiprazole, risperidone, and quetiapine.^34,36,39–41,45,46^ In four studies evaluating haloperidol at day 19 post-injury, the mean percentage of time passed in the target quadrant was similar (−3.05%; 95% CI: −13.80, 7.70), and thus no difference in memory retention was shown in Figure 3. In the two studies evaluating aripiprazole 0.1 mg/kg at day 19, the mean percentage of time passed in the target quadrant was similar compared to the vehicle group (4.74%; 95% CI: −11.57, 21.06). In the two studies evaluating risperidone 0.45 mg/kg at day 19, the mean percentage of time passed in the target quadrant was also similar compared to the vehicle group (0.08%; 95% CI: −18.43, 18.60). As for quetiapine, no differences in memory retention were observed in one study.^46^

In a mild TBI model study using a water-finding task, contrarily to the previous studies, the administration of a single dose of haloperidol and sulpiride (a D2 antagonist) significantly shortened latencies for finding and drinking, suggesting improved learning and memory deficits.47 In another study by the same authors, sulpiride improved learning and memory deficits whereas SCH-23390, a D1 antagonist, had no effects.^48^ Interestingly, the coadministration of both agents at doses that had no effect alone significantly improved learning and memory deficits.^41^ In the only study evaluating levomepromazine in a TBI model and administered 1 h before testing, the drug worsened performance in a sound conditioning experiment compared to the control group.^50^ Additionally, levomepromazine deteriorated performance in a maze learning experiment in both non-TBI and TBI animals.^50^

### Histology

A total of seven studies evaluated the effects of antipsychotics on cortical lesion size and neuron morphology.^11,33,36,39,40,42,45^ In four studies, haloperidol was not associated with increased lesion size.^39,41,42,45^ In another study, both risperidone and haloperidol were not associated with decreases in morphologically intact neurons in the ipsilateral hemisphere.^33^ In one study, aripriprazole seemed to confer neuroprotection as demonstrated by more morphologically intact CA_3_ neurons in the hippocampus ipsilateral to the impact as well as smaller cortical lesion sizes.^36^ However, these findings were not replicated in a second study, which found no difference in mean cortical sizes in the aripiprazole group compared to the vehicle group.^40^

### Effects of continuous versus intermittent dosing

One study compared the effects of continuous daily and intermittent every other day dosing of haloperidol and quetiapine in a cortical impact TBI model.^46^ Results showed no differences between intermittent and continuous groups of haloperidol and quetiapine for both motor performance tests (beam-balance and beam-walk). However, cognitive functions measured with the MWM and visual platform placement were significantly impaired in the continuous haloperidol group compared to the quetiapine and vehicle groups.^49^ A second study compared intermittent risperidone 0.45 mg/kg (one and three times a week), daily risperidone 0.45 mg/kg, and a vehicle group.^34^ The daily group exhibited significantly greater motor (beam-walk and beam-balance) and cognitive impairments (MWM) over days 1–5 post-injury compared to the vehicle group. No impairments in motor and cognitive functions were reported for the once-weekly and thrice-weekly dosing regimens.

## Discussion

This systematic review and meta-analysis evaluated the motor, cognitive, and histological effects of eight different antipsychotics administered after experimental TBI in 15 pre-clinical studies involving 1188 rodents. The systematic review and meta-analysis revealed no consistent effect of haloperidol on motor functions as evaluated by the beam-balance and beam-walk tasks. However, risperidone was associated with a significant impairment in motor function on the beam-walk task at day 5 post-injury. Other atypical antipsychotics (aripiprazole, olanzapine, and quetiapine) did not result in impaired motor function. In fact, aripiprazole was associated with improved beam-walk scores. When evaluating cognitive function, haloperidol- and risperidone-treated rats were consistently impaired when compared to the control groups. This impairment remained present with haloperidol-exposed rodents 1 month after withdrawal of the medication.^45^ However, only continuous daily administration of haloperidol and risperidone were associated with cognitive dysfunction when compared to less frequent administration.^34,46^

These results contrast with an earlier study in a mild TBI model where haloperidol and sulpiride (a D2 antagonist) improved learning and memory deficits.^47^ The use of a single drug dose post-TBI and differences in rodent species, severity of TBI, and type of model may explain these findings.^52^ In studies evaluating other atypical antipsychotics, no impairments were observed. In an early craniocervical injury model study, levomepromazine administered 1 h before testing deteriorated performance in a sound conditioning experiment.^50^ However, no other studies evaluated levomepromazine in other models or with a greater delay between the drug administration and the evaluation. Finally, we found no studies evaluating other commonly used antipsychotics such as loxapine or ziprasidone. Given that the overall risk of bias was low despite missing information regarding certain domains, we believe the results of our systematic review and meta-analysis are robust.

The negative effects of continuous haloperidol and risperidone on cognitive performance are thought to be mostly secondary to alterations in dopamine neurotransmission. Given that dopamine receptors are expressed in brain regions often affected by TBI, such as the frontal cortex and striatum, modification in dopamine transmission may have an important effect on cognition.^53^ By interfering with dopaminergic circuits, antipsychotics with D_2_ antagonist properties, such as haloperidol and risperidone, may impede processes important to cognitive recovery.^22,23^ In addition, pre-clinical studies have shown the potential for D_2_ agonists to improve functional outcomes.^54,55^ The safety of the other atypical antipsychotic agent such as aripiprazole may reside in the absence of D2 antagonism and its D2 and 5-HT1A receptor agonism.^36^ Observational studies in human TBI patients have also suggested longer post-traumatic amnesia duration with typical antipsychotics.^24,25^ Studies demonstrating the potential of amantadine, a dopamine agonist, to improve functional outcomes in TBI further supports the hypothesis of harm of dopamine receptor blockade.^26,27^

Despite data suggesting that the use of these agents is detrimental to functional recovery post-TBI, their use remains frequent.^6,56–58^ In an eight-site Canadian observational study of moderate and severe TBI in the ICU conducted in 2016, 44% of patients were exposed to antipsychotics (for a total of 26% of patient-days).^6^ Haloperidol was the most commonly used agent (28%), followed by quetiapine (26%). Similar results were reported in an Australian study, where 42% of TBI patients were administered antipsychotics during inpatient rehabilitation.^59^ In a single-center study of patients admitted from 2013 to 2018 to a TBI clinic in the United States who received psychiatric consultation for agitation/aggression, 45% of patients received a typical antipsychotic such as haloperidol^60^ whereas a Finish study of TBI patients admitted to the ICU between 2003 and 2013 found that 35% of survivors were new outpatient antipsychotic users at 1 year.^61^ Unfortunately, no large clinical studies in humans have formally studied the safety of antipsychotics in TBI.^7,20,21^

In a recent survey of Canadian intensivists, 44% of respondents believed that the use of antipsychotics for TBI-associated agitation was safe.^62^ Hence, large multi-center placebo-controlled trials evaluating the effects of both typical and atypical antipsychotics on both clinical (agitation, aggression, and rescue medication use) and safety outcomes (length of post-traumatic amnesia, cognitive function, and adverse events) are needed. Given the encouraging effects on motor function in one pre-clinical study, aripiprazole should be considered as a premier candidate.^40^ Although one case report has suggested potential improvements in cognitive and behavioral impairments post-TBI, there have been no clinical studies of aripiprazole for TBI-associated agitation in humans.^63^

The are several strengths to our systematic review and meta-analysis. First, we provided an overview of the cognitive and motor function effects of antipsychotics in TBI. We also conducted an exhaustive literature search and performed risk-of-bias assessment for all studies. There are also several limits to our systematic review and meta-analysis. First, the pre-clinical studies included in this systematic review and meta-analysis solely included rodents. Although polytrauma models with rat strains such as Sprague-Dawley are frequently used in trauma research, humans and rodents may respond differently to TBI.^52^ For example, the duration of the neuroinflammatory response to TBI is transient in rodents whereas it can last several years in humans.^52,64^ To increase transferability to humans suffering from a TBI, we chose to only include TBI models in our review, thereby excluding studies with other type of neurological injuries such as cortical trauma sensorimotor cortex injuries.^55,65,66^ Given the exploratory hypothesis-generating nature of this study, we conducted numerous analyses without adjusting for multiplicity of testing.

## Conclusion

In conclusion, there was no consistent effect of haloperidol on motor functions, but risperidone was associated with a significant impairment in motor function on day 5 post-injury. Other atypical antipsychotics did not result in impaired motor function. The continuous administration of haloperidol or risperidone may impede cognitive recovery from TBI. Whereas intermittent dosing of haloperidol and risperidone seemed safer, studies in humans are warranted. Overall, the risk of bias of the included studies was deemed to be low. Based on our findings, clinicians should avoid the continuous use of haloperidol and risperidone until human studies are available. Based on the absence of a negative impact on motor and cognitive function, aripiprazole, quetiapine, and olanzapine appear safer alternatives. Large multi-center placebo-controlled trials evaluating the effects of both typical and atypical antipsychotics on clinical and safety outcomes are strongly needed.

## Supplementary Material

Supplemental data

Supplemental data

## Abbreviations Used

CI: confidence interval
ICU: intensive care unit
MWM: Morris water maze
PRISMA: Preferred Reporting Items for Systematic Reviews and Meta-Analyses
SYRCLE: Systematic Review Centre for Laboratory Animal Experimentation
TBI: traumatic brain injury
