# Supplementary material for: Cognitive and Motor Function Effects of Antipsychotics in Traumatic Brain Injury: A Systematic Review of Pre-Clinical Studies
Source: Neurotrauma Rep. 2024 Mar 5;5(1):181–93. doi: 10.1089/neur.2023.0108 (PMC10924062; doi:10.1089/neur.2023.0108)
Supplement: Supplemental data [file Suppl_FigureS1.docx]

Supplement 1 – Medline search strategy

| 1. | exp Craniocerebral Trauma/ | | | | | | |  |  |  |  |  |
| --- | --- | --- | --- | --- | --- | --- | --- | --- | --- | --- | --- | --- |
| 2. | (Craniocerebral Traumas or Craniocerebral Trauma or Craniocerebral injury or Craniocerebral injuries or Head Injury or Head Injuries or head trauma or head traumas or Parietal Region Trauma or Parietal Region Traumas or Skull Injury or Skull Injuries or Head Injury or Head Injuries or Occipital Region Trauma or Occipital Region Traumas or Occipital Trauma or Occipital Traumas or Temporal Region Trauma or Temporal Region Traumas or Frontal Region Trauma or Frontal Region Traumas or Forehead Trauma or Forehead Traumas or Brain Concussion or Brain Concussions or Diffuse Axonal Injury or Diffuse Axonal Injuries).mp. | | | | | | | | | |  |  |
| 3. | (Traumatic Intracranial Hemorrhage or Traumatic Intracranial Hemorrhages or Traumatic Intracranial Hematoma or Traumatic Intracranial Hematomas or Glasgow Coma scale or Brain Damage or Brain Damages).mp. | | | | | | | | | |  |  |
| 4. | exp Brain Damage, Chronic/ | | | |  |  |  |  |  |  |  |  |
| 5. | 1 or 2 or 3 or 4 |  |  |  |  |  |  |  |  |  |  |  |

| 6. | exp Cognition/ or Neurocognitive Disorders/ or exp Amnesia/ or exp Cognition Disorders/ | | | | | | | | | | |  |  |
| --- | --- | --- | --- | --- | --- | --- | --- | --- | --- | --- | --- | --- | --- |
| 7. | (Cognition or Cognitions or Cognitive or Neurocognitive Disorder or Neurocognitive Disorders or amnesia or amnesias or amnesic).mp. | | | | | | | | | | |  |  |
| 8. | Auditory Perceptual Disorders/ or exp Space Perception/ or exp Memory/ or Repetition Priming/ or exp Proprioception/ or Kinesthesis/ or Maze Learning/ or exp Spatial Learning/ | | | | | | | | | | |  |  |
| 9. | (Auditory Perceptual Disorder or Auditory Perceptual Disorders or Auditory Processing Disorder or Auditory Processing Disorders or Psychoacoustical Disorder or Psychoacoustical Disorders or Auditory Comprehension Disorder or Auditory Comprehension Disorders or Space Perception or Space Perceptions or memory or Repetition Priming or Proprioception or Proprioceptions or Position Sense or Kinestheses or Kinesthesis or Kinesthesia or Movement Sensation or Movement Sensations or Maze Learning or Spatial Learning).mp. | | | | | | | | | | |  |  |
| 10. | | exp Consciousness Disorders/ or exp Unconsciousness/ or Stupor/ | | | | | | |  |  |  |  |  |
| 11. | | (Consciousness or Semiconsciousness or Unconsciousness or stupor or narcosis).mp. | | | | | | | | | |  |  |
| 12. | | Recovery of Function/ | | | |  |  |  |  |  |  |  |  |
| 13. | | (Function Recovery or Function Recoveries or Recovery of Function or Recovery of Functions).mp. | | | | | | | | | |  |  |
| 14. | | 6 or 7 or 8 or 9 or 10 or 11 or 12 or 13 |  |  |  |  |  |  |  |  |  |  |  |

| 15. | Behavior, Animal/ or Escape Reaction/ or Translational Medical Research/ |  |  |  |  |  |
| --- | --- | --- | --- | --- | --- | --- |
| 16. | (Animal Behavior or Animal Behaviors or Animal Behaviour or Animal Behaviours or Flight Reaction or Flight Reactions or Escape Reaction or Escape Reactions or Translational Medicine or Translational Medical Research or Translational Biomedical Research ORTranslational Research or Translational Medical Science or Knowledge Translation or Knowledge Translations).mp. | | | |  |  |

| 17. | 15 or 16 | | | |  |  |  |  |  |
| --- | --- | --- | --- | --- | --- | --- | --- | --- | --- |
| 18. | exp Antipsychotic Agents/ or Antipsychotic/ or Major Tranquilizer/ or Major Tranquillizing/ or exp Tranquilizing Agents/ | | | | | | |  |  |
| 19. | (acepromazine or amoxapine or asenapine or azaperone or benperidol or butaclamol or chlorpromazine or chlorprothixene or clopenthixol or clozapine or droperidol or flupenthixol or fluphenazine or fluspirilene or haloperidol or levomepromazine or loxapine or loxapine succinate or mesoridazine or methiothepin or methotrimeprazine or molindone or olanzapine or paliperidone or penfluridol or perazine or perphenazine or pimozide or prochlorperazine or promazine or quetiapine or remoxipride or reserpine or risperidone or ritanserin or spiperone or sulpiride or thioridazine or thiothixene or tiapride hydrochloride or trifluoperazine or trifluperidol or triflupromazine or ziprasidone).mp. | | | | | | |  |  |
| 20. | 18 or 19 |  |  |  |  |  |  |  |  |

| 21. | 5 and 14 and 20 |  |  |
| --- | --- | --- | --- |

| 22. | 5 and 17 and 20 |  |  |
| --- | --- | --- | --- |

| 23. | 21 or 22 |
| --- | --- |
