# Supplementary material for: Cognitive and Motor Function Effects of Antipsychotics in Traumatic Brain Injury: A Systematic Review of Pre-Clinical Studies
Source: Neurotrauma Rep. 2024 Mar 5;5(1):181–93. doi: 10.1089/neur.2023.0108 (PMC10924062; doi:10.1089/neur.2023.0108)
Supplement: Supplemental data [file Suppl_TableS1.docx]

<TT>**Supplementary Table S1. Study Quality and SYRCLE’s Risk of Bias Assessment**</TT>

|  | Selection bias | Selection bias | Selection bias | Performance bias | Performance bias | Detection bias | Detection bias | Attrition bias | Reporting bias | Other |
| --- | --- | --- | --- | --- | --- | --- | --- | --- | --- | --- |
| Study (1st Author, Year) | 1. Was the allocation sequence adequately generated & applied? | 2. Were the groups similar at baseline or were they adjusted for confounders in the analysis? | 3. Was the allocation adequately concealed? | 4. Were the animals randomly housed during the experiment? | 5. Were the caregivers and/or investigators blinded from knowledge which intervention each animal received during the experiment? | 6. Were animals selected at random for outcome assessment? | 7. Was the outcome assessor blinded? | 8. Were incomplete outcome data adequately addressed? | 9. Are reports of the study free of selective outcome reporting? | 10. Was the study apparently free of other problems that could result in high risk of bias? |
| Bao et al.  2019 | Unclear | Yes | Unclear | Yes | Yes | Unclear | Yes | Yes | Yes | Yes |
| Besagar et al. 2019 | Unclear | Yes | Unclear | Yes | Yes | Unclear | Yes | Yes | Yes | Yes |
| Boismare et al. 1978 | No | Unclear | Unclear | Unclear | Unclear | Unclear | Unclear | Unclear | Yes | No |
| Carlson et al 2018 | Unclear | Yes | Unclear | Yes | Yes | Unclear | Yes | Yes | Yes | Yes |
| Folweiler et al 2017 | Unclear | Yes | Unclear | Yes | Yes | Unclear | Yes | Yes | Yes | Yes |
| Free et al. 2017 | Unclear | Yes | Unclear | Yes | Yes | Unclear | Yes | Yes | Yes | Yes |
| Hoffman et al. 2008 | Unclear | Yes | Unclear | Yes | Yes | Unclear | Yes | Yes | Yes | Yes |
| Kline et al. 2007 | Unclear | Yes | Unclear | Yes | Yes | Unclear | Yes | Yes | Yes | Yes |
| Kline et al. 2008 | Unclear | Yes | Unclear | Yes | Yes | Unclear | Yes | Yes | No | Yes |
| Phelps et al. 2015 | Unclear | Yes | Unclear | Yes | Yes | Unclear | Yes | Yes | Yes | Yes |
| Phelps et al. 2017 | Unclear | Yes | Unclear | Yes | Yes | Unclear | Yes | Yes | Yes | Yes |
| Tang et al.  1997 (a) | Unclear | Yes | Unclear | Yes | Yes | Unclear | Yes | Yes | Yes | Yes |
| Tang et al.  1997 (b) | No | Unclear | Unclear | Unclear | Unclear | Unclear | Unclear | Unclear | Yes | No |
| Weeks et al. 2018 | Unclear | Yes | Unclear | Yes | Yes | Unclear | Yes | Yes | Yes | Yes |
| Wilson et al. 2003 | Unclear | Unclear | Unclear | Yes | Unclear | Unclear | Unclear | No | Yes | No |
